# Supplementary material for: Dynamic transcriptomic profiles of zebrafish gills in response to zinc supplementation
Source: BMC Genomics. 2010 Oct 11;11:553. doi: 10.1186/1471-2164-11-553 (PMC3091702; doi:10.1186/1471-2164-11-553)
Supplement: Additional file 2 — Interactive Direct Interaction Network representing the molecular interactions between zinc, copper, iron, calcium and proteins encoded by transcripts changed by zinc supplementation. Mini web-site containing index.html and hyperlinked pages in subdirectory describing a Direct Interaction Network automatically generated based on curated interactions contained within the proprietary PathwayArchitect database. Ovals represent proteins and the circles symbolize metal ions. Objects are coloured by their abundance in zebrafish at the time-point they were significantly different from the control is a scale from -4 fold (dark green) to +4 fold (dark red). Where significant differences were found at more than one time-point, the colour overlay shows expression at the first instance. Dark blue squares denote 'binding', and light blue squares 'expression'; green squares stand for 'regulation', green diamonds for 'metabolism', and green circles for 'promoter binding'. Arrow heads indicate directionality of the interaction where annotated. All nodes and edges can be further interrogated by selecting the relative area of the image. [file 1471-2164-11-553-S2.zip › PathwayArchitect Zn xs DIN/395278.html]

# METABOLISM:

|  |  |
| --- | --- |
| Type | METABOLISM |
| Effect | None |


---

|  |  |
| --- | --- |
| Score | 0 |


---

|  |  |
| --- | --- |
| Reference Count | 8 |


---

|  |  |
| --- | --- |
| Mechanism | Unknown |


---

|  |  |
| --- | --- |
| Reference:0 || Sentence | "These results indicate that the induction of metallothionein synthesis in lymphoblastoid cells is controlled by the level of intracellular copper, suggesting that the major defect in Menkes' cells is not due to the abnormal regulation of metallothionein synthesis but to an alteration of the copper metabolism in cells by which the levels of intracellular copper become larger than those in normal cells and just lower than the threshold level for induction of metallothionein synthesis." |
| PMID | 3494730 |
| Year | 1987 |
| Species | Human |
| Journal | J Biol Chem |
| RefScore | 3 |
| Source | PArchNLP |
  |
|  |  |


---

|  |  |
| --- | --- |
 Reference:1 || Sentence | "It appears that the elevated levels of copper and zinc, rather than gold, are responsible for the induction of MT synthesis." |
| PMID | 3118513 |
| Year | 1987 |
| Species | Rat |
| Journal | Toxicology |
| RefScore | 1 |
| Source | PArchNLP |
  ||  |  |


---

|  |  |
| --- | --- |
 Reference:2 || Sentence | "To clarify the physiological roles of heat shock proteins induced by copper, we studied the synthesis of these proteins and metallothionein, as well as the level and nature of copper incorporated into HeLa cells." |
| PMID | 1783602 |
| Year | 1991 |
| Species | Human |
| Journal | J Biochem (Tokyo) |
| RefScore | 1 |
| Source | PArchNLP |
  ||  |  |


---

|  |  |
| --- | --- |
 Reference:3 || Sentence | "These alterations include 1) decreased skeletal muscle accretion due to increased rates of protein degradation and decreased protein synthesis; 2) increased basal metabolic rate resulting in increased energy utilization; 3) use of dietary amino acids for gluconeogenesis and as an energy source instead of for muscle protein accretion; 4) synthesis by the liver of acute phase proteins; 5) redistribution of iron, zinc, and copper within the body due to the hepatic synthesis of metallothionein, ferritin, and ceruloplasmin; (6) impaired accretion of cartilage and bone; and 7) release of hormones such as insulin, glucagon, and corticosterone." |
| PMID | 1717968 |
| Year | 1991 |
| Species | Human |
| Journal | Poult Sci |
| RefScore | 2 |
| Source | PArchNLP |
  ||  |  |


---

|  |  |
| --- | --- |
 Reference:4 || Sentence | "Model 2 is an impaired renal copper reabsorption resulting in a toxic intracellular copper concentration which induces metallothionein synthesis to sequester copper." |
| PMID | 7188777 |
| Year | 1980 |
| Species | Mouse |
|  | Human |
| Journal | J Nutr |
| RefScore | 2 |
| Source | PArchNLP |
  ||  |  |


---

|  |  |
| --- | --- |
 Reference:5 || Sentence | "The data suggest that the accumulation of copper induces synthesis of cytoplasmic and nuclear metallothionein, and that the increased amount of cytoplasmic copper is bound to a similarly increased amount of metallothionein." |
| PMID | 6727583 |
| Year | 1984 |
| Species | Human |
|  | Rat |
| Journal | Liver |
| RefScore | 1 |
| Source | PArchNLP |
  ||  |  |


---

|  |  |
| --- | --- |
 Reference:6 || Sentence | "The altered metabolism of hepatic MT in diabetic rats was attributed primarily to disturbances in endocrine status, while the altered metabolism of renal MT was largely due to accumulation of excessive dietary copper in the kidney." |
| PMID | 3409670 |
| Year | 1988 |
| Species | Rat |
| Journal | Comp Biochem Physiol B |
| RefScore | 1 |
| Source | PArchNLP |
  ||  |  |


---

|  |  |
| --- | --- |
 Reference:7 || Sentence | "Effects of metallothionein (MT) synthesis inhibiting compounds (actinomycin D, cycloheximide), MT synthesis stimulating compounds (dexamethasone, dibu-cAMP) and interfering metals (Cd, Zn) on copper accumulation were investigated in rat hepatoma tissue culture cells." |
| PMID | 7940570 |
| Year | 1994 |
| Species | Rat |
| Journal | Toxicology |
| RefScore | 1 |
| Source | PArchNLP |
  |


---

|  |  |
| --- | --- |
